# A PI Control Method with HGSO Parameter Regulator for Trajectory Planning of 9-DOF Redundant Manipulator

**DOI:** 10.3390/s22186860

**Published:** 2022-09-10

**Authors:** Meijiao Liu, Tianyu Liu, Mingchao Zhu, Liheng Chen

**Affiliations:** 1 CAS Key Laboratory of On-Orbit Manufacturing and Integration for Space Optics System, Changchun Institute of Optics, Fine Mechanics and Physics, Chinese Academy of Sciences, Changchun 130033, China; 2 School of Optoelectronics, University of Chinese Academy of Sciences, Beijing 100049, China

**Keywords:** 9-DOF redundant manipulator, trajectory planning, HGSO, PI control, parameter adjustment

## Abstract

In order to solve the tracking accuracy problem of the redundant manipulator, a PI control method with Henry gas solubility optimization parameter regulator (PI-HGSO) is proposed in this paper. This method consists of the controller and the parameter regulator. The characteristic is that the position deviation of a manipulator is equivalent to a specific function; namely, the proportional-integral (PI) controller is used to adjust the deviation input. The error can be better corrected by the processing of the PI controller so that the inverse kinematics solution of the minimum error can be realized. At the same time, the parameter selection of PI controllers has always been a difficulty in controller design. To address the problem, Henry gas solubility optimization (HGSO) is selected as a parameter regulator to optimize the parameters and obtain the optimal controller, thereby achieving high-precision trajectory tracking. Experiments on 9-DOF redundant manipulator show that our method achieves competitive tracking accuracy in contrast with others. Meanwhile, the efficiency and accuracy of the PI controller are greatly guaranteed by using HGSO to automatically optimize controller parameters instead of making approximate adjustments through infinite manual trial and error. Therefore, the feasibility and competitive superiority of PI-HGSO is fully proved in trajectory planning of redundant manipulators.

## 1. Introduction

With the development of technology and industry, redundant manipulator that can achieve high flexibility and high precision is gradually developed to meet the needs of modern intelligent industry. Although the redundant manipulator can complete more complex tasks, it is more difficult to solve the problems of related kinematics planning and control. Trajectory planning of redundant manipulators includes planning a set of end trajectories and completing the movement of the end trajectory under the manipulator’s limited conditions of the tracking accuracy, joint velocity, and joint angle during the movement.

Trajectory planning methods of the redundant manipulator are mainly divided into the geometric method, iterative method, and numerical method [1,2,3,4,5,6,7,8,9]. The geometric method is to analyze each joint angle according to the geometric relationship between the end posture and the specific angles in sequence. However, it is only applicable to manipulators with simple or special structures. The iterative method is to solve the inverse kinematics solution iteratively by an intelligent algorithm. Nevertheless, the iterative method is used to solve the joint angles corresponding to each pose, the computation is heavy, and the real-time performance is affected. At the same time, the traditional intelligent optimization algorithms, including a genetic algorithm (GA) [10,11,12], ant colony optimization (ACO) [13,14], particle swarm optimization (PSO) [15,16], grey wolf optimizer (GWO) [17,18,19,20], etc., have certain shortcomings, such as consistent initial value setting, slow initial optimization speed, complex algorithm structure, slow convergence speed, and easy to fall into the local optimal. The algebraic numerical method is to calculate joint angles by the Jacobian matrix, such as the least squares method, gradient projection method [21,22,23], etc. The algebraic method is a universal method, but the parameter value has a great impact on the accuracy. Usually, it is not easy to find the appropriate parameter value.

In this paper, a PI control method with an HGSO parameter regulator (PI-HGSO) is proposed to perform trajectory planning f a 9-DOF redundant manipulator. Proportional-integral (PI) controller is designed to set the optimum joint angles from the current to the desired end pose. Meanwhile, Henry gas solubility optimization (HGSO) is used to solve the appropriate controller parameters to achieve the trajectory planning of the redundant manipulator. PI controller [5,24,25,26] has strong model identification ability and can more accurately equivalent various functions. In addition, the PI controller is simple in structure and easy to use to give a value close to the deviation that needs to be assigned to the end-effector. Therefore, the PI controller is selected to deal with the deviation of the end to the expected pose to achieve motion control. Traditional parameter tuning relies on empirical trial and error with low efficiency and accuracy. At the same time, the introduction of intelligent algorithms for parameter adjustment can automatically achieve efficient and accurate parameter selection. HGSO is based on the principle of gas solubility function to design the optimization algorithm [27,28,29,30,31]. The algorithm has not only a strong optimization ability but also groups the swarm particles to achieve multi-group parallel optimization. This characteristic improves the speed of optimization and effectively overcomes the problem of local optimum. Hence, HGSO is selected to optimize the PI controller parameters to further improve the accuracy of the motion planning.

The specific contributions of the PI control method with HGSO parameter regulator (PI-HGSO) for trajectory planning of 9-DOF redundant manipulator can be summarized as follows:

- The proposed PI-HGSO method enables the designed PI controller to obtain accurate gains during the trajectory planning process for redundant manipulators, achieving a highly precise position tracking of the end-effector.
- The design can obtain PI controller parameters more quickly and efficiently by using the HGSO parameter regulator instead of the traditional empirical trial and error, improving the controller parameter tuning cycle.
- The PI controller is used to replace the traditional gain compensation system to achieve the end-effector position compensation of the deviation part efficiently. The design structure is simple, and the error is effectively suppressed. Besides, the feasibility of the design has been guaranteed since this paper has proved that PI-HGSO is asymptotically stable in the Cartesian deviation trajectory of the endpoint.

The structure of the rest is organized as follows: the second part describes the PI controller with joint limits for trajectory planning; the third part describes the design of parameter regulator by HGSO; the fourth part completes the simulation and results of analysis under the 9-DOF redundant manipulator; the fifth part is conclusion and summary.

## 2. PI Controller with Joint Limits for Trajectory Planning of Redundant Manipulator

**Figure 1:**
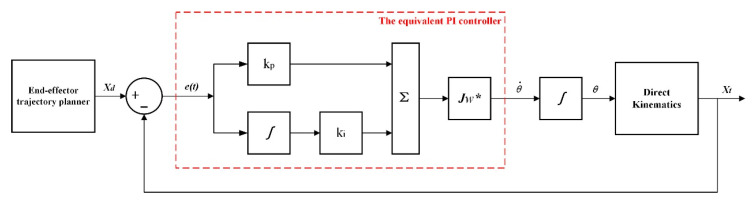
The block diagram of the path planning method that is equivalent to a PI controller.

In order to solve the inverse kinematics of the redundant manipulator, the algebraic method needs to calculate the joint angles through the pose deviation. The deviation calculation of the end-effector needs to satisfy a certain functional relationship, and this characteristic has a strong fit with the PI characteristic. Therefore, in this paper, the expected path planning of the end-effector is equivalent to a PI controller [5,24,26]. The schematic diagram is shown in Figure 1, and the equivalent formula is described as:

(1) $$\delta =k_{p}(x_{d}-x_{t})+k_{i}\int_{0}^{t}(x_{d}-x_{t})dt$$

where δ is defined to be the PI controller as the deviation expected to be given at the end-effector; $\left(x_{d}-x_{t}\right)$ is the difference between the expected position and the current position; $k_{p}$ and $k_{i}$ are the PI controller parameters.

**Theorem** **1.** *The stability of the equivalent PI controller method can be proved by the Lyapunov stability theorem [5,25,32].*

We denote the end-Cartesian space deviation by $e\left(t\right)=x_{d}-x_{t}$, where $\dot{e}\left(t\right)$ represents its time derivative. The speed of position deviation can be compensated by designing a PI linear system. Thus, it is written as $\dot{e}\left(t\right)=-\delta$, we have:

(2) $$\dot{e}\left(t\right)=-\delta =-k_{p}e\left(t\right)-k_{i}\int_{0}^{t}e\left(t\right)dt$$

We define the Lyapunov function as:

(3) $$v\left(t\right)=e^{2}\left(t\right)+k_{i}{\left(\int_{0}^{t}e\left(t\right)dt\right)}^{2}$$

Which is obtain v(t)≥0, and the positive definiteness of v(t) is guaranteed. For the defined Lyapunov function v(t), we can obtain the time derivative $\dot{v}\left(t\right)$, which is derived as:

(4) $$\begin{array}{ll} \dot{v}\left(t\right) & =2e\left(t\right)\dot{e}\left(t\right)+2k_{i}e\left(t\right)\int_{0}^{t}e\left(t\right)dt\\ & =2e\left(t\right)\left(-k_{p}e\left(t\right)-k_{i}\int_{0}^{t}e\left(t\right)dt\right)+2k_{i}e\left(t\right)\int_{0}^{t}e\left(t\right)d\\ & =-2k_{p}e^{2}\left(t\right)\leq 0 \end{array}$$

where $\dot{v}\left(t\right)\leq 0$, it can be guaranteed the negative definiteness of $\dot{v}\left(t\right)$. According to the Lyapunov theory, the trajectory of the end-Cartesian space deviation e(t) is asymptotically stable in the equivalent PI controller method.

In the inverse kinematics solution process of the redundant robot, the value that needs to be acted on the end-effector is mapped to the joint space through the Jacobian matrix. Whereupon, the velocity of joint space is obtained, which is expressed as:

(5) $$\dot{\theta }=J^{-1}\delta$$

Minimum norm method with weighting coefficient [9,33,34,35] was proposed to solve the redundant system problem. Its basic idea is to use the minimum weighted norm by adding the weighted matrix to realize the task limitation. In order to satisfy the joint angle constraint and reduce the joint angular velocity, the Jacobian matrix optimized by minimum norm with weighting coefficient is usually used. When the joint angular velocity is too large, the weighted coefficient is increased as a penalty factor to reduce the joint velocity and achieve the restriction of joint velocity.

The Jacobian matrix with weighting coefficient is expressed as:

(6) $$J_{w}=JW$$

(7) $$W=diag\left(w_{1},w_{2},\cdots ,w_{i},\cdots w_{9}\right)$$

(8) $$w_{i}=\{\begin{array}{cc} 1+\left|\frac{\partial H\left(\theta \right)}{\partial \theta_{i}}\right| & \;if\;\Delta \left|\frac{\partial H\left(\theta \right)}{\partial \theta_{i}}\right|\geq 0\\ 1 & \;if\;\Delta \left|\frac{\partial H\left(\theta \right)}{\partial \theta_{i}}\right|<0 \end{array}$$

where J is the Jacobian matrix, $J_{w}$ is the weighted Jacobian, W is the weighted matrix, and H(θ) is the constructed objective function.

To avoid approaching the joint limit, the objective optimization function H(θ) is defined as:

(9) $$H\left(\theta \right)=\frac{1}{9}\sum_{i=1}^{9}\frac{{\left(\theta_{i,\max}-\theta_{i,\min}\right)}^{2}}{\left(\theta_{i,\max}-\theta_{i}\right)\left(\theta_{i}-\theta_{i,\min}\right)}$$

(10) $$\frac{\partial H\left(\theta \right)}{\partial \theta_{i}}=\frac{1}{9}\sum_{i=1}^{9}\frac{{\left(\theta_{i,\max}-\theta_{i,\min}\right)}^{2}\left(2\theta_{i}-\theta_{i,\max}-\theta_{i,\min}\right)}{{\left(\theta_{i,\max}-\theta_{i}\right)}^{2}{\left(\theta_{i}-\theta_{i,\min}\right)}^{2}}$$

**Figure 2:**
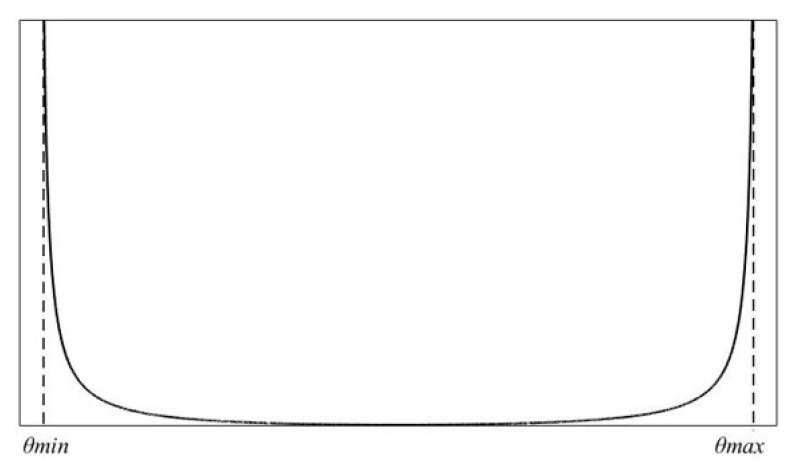
The graph of the objective function.

Figure 2 is the graph of the objective function. It can be seen from Figure 2 that when the joint angle approaches the joint limit, the designed objective optimization function H(θ) approaches infinity, and the value of $\frac{\partial H\left(\theta \right)}{\partial \theta_{i}}$ also approaches infinity. Moreover, H(θ) and $\frac{\partial H\left(\theta \right)}{\partial \theta_{i}}$ are almost 0 within the joint limit range. Therefore, in order to avoid the joint angle exceeding the limit, the smaller the value of the optimization function the better. When the joint angle changes to the limit value, the joint velocity will be reduced by increasing the weight coefficient. Therefore, the restriction on the joint angle will be implemented.

For the redundant manipulator, its Jacobian matrix is a long square matrix, and the inverse cannot be calculated. So, the pseudo-inverse can be used. However, the pseudo-inverse of the Jacobian matrix [9,36,37] will lead to the problem of matrix singularity. In order to avoid the singularity, the Jacobian inverse with singularity robustness is introduced, and its formula is described as:

(11) $$J^{*}=J^{T}{\left(JJ^{T}+\lambda I\right)}^{-1}$$

The Jacobian inverse with singularity robustness and weighting coefficient is expressed as:

(12) $$J_{w}^{*}=W^{-1}J^{T}{\left(JW^{-1}J^{T}+\lambda I\right)}^{-1}$$

Thus, the inverse kinematics solution of using the weighted minimum norm method is expressed as:

(13) $$\dot{\theta }=J_{w}^{*}\delta =W^{-1}J^{T}{\left(JW^{-1}J^{T}+\lambda I\right)}^{-1}\delta$$

By combining the weighted Jacobian minimum norm and singularity robustness inverse, the joint velocity norm can be reduced by $J_{w}^{*}$. Therefore, this will play a role in planning the joint velocity to some extent. And I am going to give λ a small enough value to avoid the singularities.

## 3. A PI Control Method with HGSO Parameter Regulator

**Figure 3:**
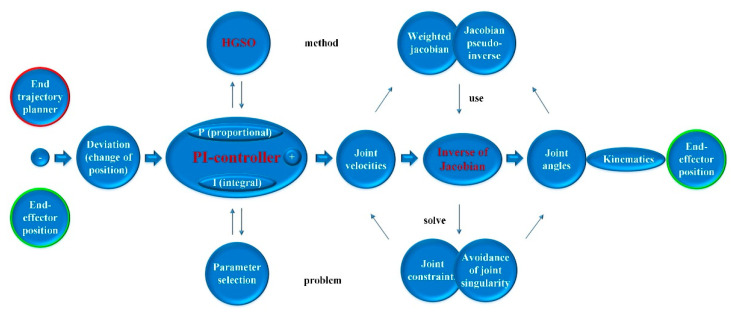
The specific scheme structure diagram of PI-HGSO.

For the joint angle constraint problem and joint angle singularity problem, the Jacobian pseudoinverse with weight coefficient is applied to complete the transformation of joint angular velocity and joint angle. At the same time, in order to solve the problem of manual selection of PI controller parameters by experience, HGSO is selected to optimize PI parameters, ensuring the requirement of a small error value in the process of redundant manipulator trajectory planning. Thereupon, A PI control method with an HGSO parameter regulator is designed to perform trajectory planning of the 9-DOF redundant manipulator, and the specific scheme is presented in Figure 3.

### 3.1. Introduction and Description of HGSO

**Figure 4:**
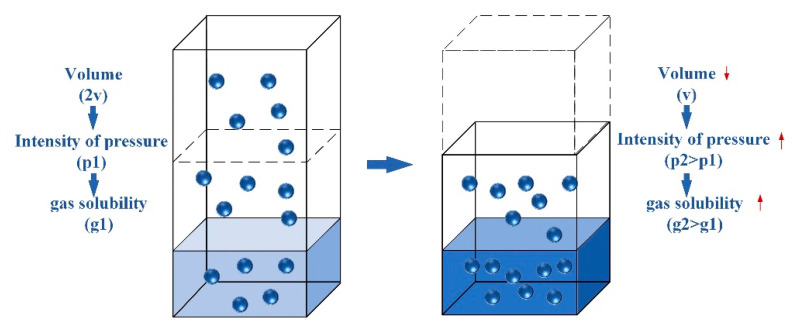
The relationship between the amount of dissolved gas particles and pressure.

A novel physics-based algorithm named Henry gas solubility optimization (HGSO) was proposed by Fatma et al. in 2019 [27]. Furthermore, the optimization performance is better than other algorithms and successfully applied to the related optimization problems. HGSO was inspired by Henry’s Gas Law, formulated by William Henry in 1803 [38]. The basic idea of Henry’s law of gas solubility is described as [27,28,29]: at a constant temperature and pressure, the solubility of a gas in a given volume of liquid is proportional to the partial pressure of the gas in equilibrium. So, temperature and pressure are going to affect the gas solubility. The schematic diagram is shown in Figure 4. The gas law is expressed by the following equation:

(14) $$S_{g}=H\times P_{g}$$

where $S_{g}$ is the gas solubility, H is Henry’s constant, $P_{g}$ is the gas partial pressure.

The Henry’s constant H is expressed as:

(15) $$H\left(T\right)=H^{\theta }\times \exp\left(\frac{-\nabla_{sol}E}{R}\left(1/T-1/T^{\theta }\right)\right)$$

where, T and $T^{\theta }$ are, respectively, the current temperature and reference temperature, $H^{\theta }$ is the reference Henry constant, R is the gas constant, and the enthalpy of dissolution is denoted by $\nabla_{sol}E$.

$\frac{\nabla_{sol}E}{R}$ is the van’t Hoff formula, and is defined as a constant C. Therefore, the Henry’s constant H is reformulated as:

(16) $$H\left(T\right)=H^{\theta }\times \exp\left(-C\times \left(1/T-1/T^{\theta }\right)\right)$$

The HGSO is established on the basis of Henry’s law of gas solubility, and related mathematical modeling formulas and steps are presented as follows:

Step 1: Initialization

At the beginning of the HGSO algorithm, N gas particles are randomly initialized to their gas positions, the initialization is based on the formula:

(17) $$X_{i}\left(t+1\right)=X_{\min}+r\times \left(X_{\max}-X_{\min}\right)$$

where $X_{i}$ is described as the position of the ith gas in population N, $X_{\max}$ and $X_{\min}$ are, respectively, the upper and lower limits of position, r is a random constant that can generate randomly the number between 0 and 1, t is the iteration time.

Furthermore, gas i is segmented into type cluster j and the other initial parameter values related to Henry’s law are expressed as:

(18) $$\begin{array}{l} H_{j}\left(t\right)=l_{1}\times rand\left(0,1\right)\\ P_{i,j}=l_{2}\times rand\left(0,1\right)\\ C_{j}=l_{3}\times rand\left(0,1\right) \end{array}$$

where $H_{j}\left(t\right)$ is the Henry’s constant of cluster j, $P_{i,j}$ is the particle pressure of gas i in cluster j, $C_{j}$ is the van’t Hoff formula value of cluster j. Among them, $l_{1}=5\times 10^{-2}$, $l_{2}=100$, $l_{3}=1\times 10^{-2}$.

Step 2: Clustering

There are M type clusters in population N, gas population needs to be divided into different clusters. So, the different Henry’s constants $H_{1},\cdots ,H_{j},\cdots H_{M}$ of M clusters are obtained.

Step 3: Fitness evaluation

According to the evaluation index function, the gas particles in each cluster j are evaluated, and the best gas particles are identified. At the same time, the best gas particles in all kinds of clusters are compared to select as the best gas particle in the whole population.

Step 4: Update Henry’s coefficient

The updating formula of Henry’s constant can be described as:

(19) $$\begin{array}{l} H_{j}\left(t+1\right)=H_{j}\left(t\right)\times \exp\left(-C_{j}\times \left(1/T\left(t\right)-1/T^{\theta }\right)\right)\\ T\left(t\right)=\exp\left(-t/t_{iter}\right),T^{\theta }=298.15 \end{array}$$

where $t_{iter}$ is whole iterations, T(t) is the temperature of iteration t.

Step 5: Update gas solubility.

The gas solubility $S_{i,j}\left(t\right)$ needed to be updated and the formula is as:

(20) $$S_{i,j}\left(t\right)=K\times H_{j}\left(t+1\right)\times P_{i,j}\left(t\right)$$

where $S_{i,j}\left(t\right)$ is the current solubility of gas i in cluster j, $P_{i,j}\left(t\right)$ is the current pressure of gas i in cluster j, K is the constant and usually defined as 1.

Step 6: Update position

The relevant formula of gas position update strategy is as follows:

(21) $$\begin{array}{l} X_{i,j}\left(t+1\right)=X_{i,j}\left(t\right)+F\times r\times \gamma \times \left(X_{j,best}\left(t\right)-X_{i,j}\left(t\right)\right)+F\times r\times \alpha \times \left(S_{i,j}\left(t\right)\times X_{best}\left(t\right)-X_{i,j}\left(t\right)\right)\\ \gamma =\beta \times \exp\left(-\frac{F_{best}\left(t\right)+\varepsilon }{F_{i,j}\left(t\right)+\varepsilon }\right),\varepsilon =0.05 \end{array}$$

where $X_{i,j}$ is the position of gas i in cluster j, $X_{j,best}$ is the best gas in cluster j, $X_{best}$ is the current best gas in the whole population, F is the function that can change the searching direction to achieve diversity and it is denoted by 1 or −1, r is a random generator that can obtain a random number between 0 and 1, α represents the influencing factor of the other gases to gas i in cluster j and defined as 1, γ is the interaction ability between gas i and other gases in cluster j, β is a constant, $F_{i,j}$ is the fitness of gas i in cluster j, and $F_{best}\left(t\right)$ is the fitness of the best gas in the whole population.

Step 7: Escape from local optimum and reset the position of the worst agents

In order to avoid the algorithm falling into the local optimum, the total particles are sorted according to the fitness value and the number of the worst particles is defined. Then these worst particles are selected, and their positions are reset. The position reset strategy is shown in Formula (21), and the relevant formula of the worst particle number is denoted as:

(22) $$N_{w}=N\times \left(rand\left(c_{2}-c_{1}\right)+c_{1}\right),\;c_{1}=0.1\;and\;c_{2}=0.2$$

where $c_{1}$ and $c_{2}$ are constants about rough worst particle proportions, $N_{w}$ is the number of the worst particles in the whole population.

### 3.2. HGSO Parameter Regulator for PI Controller

**Figure 5:**
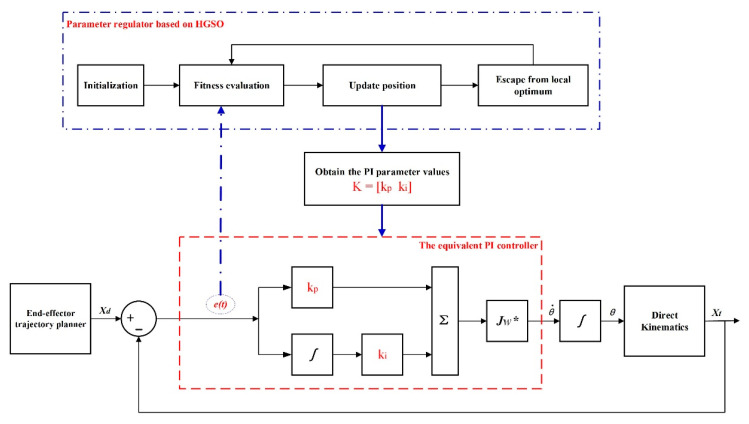
The schematic diagram of PI-HGSO for trajectory planning.

Traditionally, the parameter selection of the PI controller is mainly based on the experience and the trend of the result to adjust and choose the approximate value [24,25,26]. The method not only fails to find the appropriate value but also wastes a lot of time in adjusting parameters. Due to the optimization ability of HGSO being better than other intelligent algorithms, HGSO is chosen as a parameter adjuster to optimize the parameters of the PI controller to achieve high-precision trajectory tracking and planning [24,27,28,29,30,31]. The specific method flow is shown in Figure 5.

K is defined as the PI control parameter to be optimized, including the proportional parameter $k_{p}$ and the integral parameter $k_{i}$. The parameter K is described as:

(23) $$K=\left[k_{p}\;k_{i}\right]$$

The sum of the deviations between the actual and the expected trajectory points, is designed as the evaluation index, and the formula of the evaluation function is expressed as:

(24) $$F_{fitness}=\frac{1}{n}{\sum_{i=1}^{n}\left(x_{e\left(i\right)}^{2}+y_{e\left(i\right)}^{2}+z_{e\left(i\right)}^{2}\right)}^{1/2}$$

The main principles of PI control method based on HGSO parameter adjuster for trajectory planning are as follows: Firstly, PI parameter K was set and optimized by HGSO. Next, the calculation of the joint angle and position is completed, and the fitness $F_{fitness}$ is obtained by the evaluation function. Therefore, a better K is selected according to the evaluation index. Then, the new K is obtained through the updating mechanism of HGSO, and the parameter regulator will continue to evaluate and select the better K by HGSO. Finally, when the iteration ends, the optimized K is obtained to solve the trajectory tracking. The specific steps are as follows:

Step 1: Initialization

Initialize population particle size N, the number of type clusters M, the particle position information $X_{i}$ that is the variable K of PI controller to be optimized, and other parameters, $H_{j}$, $P_{i,j}$, $C_{j}$, etc. (i=1,⋯,N; j=1,⋯M).

Step 2: The setting of PI controller parameters:

The position information $X_{i}$ obtained by HGSO, namely PI parameter K, is assigned to the PI controller for solving joint angles in trajectory planning.

Step 3: Evaluation and update:

Complete the evaluation of the current position $X_{i}$ of variable K by the fitness function Equation (23). Afterward, according to Equation (20), the position information $X_{i}$ of the variable K is updated through HGSO’s update mechanism.

Step 4: Update the worst particles:

Select the poor particles according to Equation (21) by fitness $F_{fitness}$, meanwhile, reset the position information of the worst particles to avoid local optimality.

Step 5: Repetition and iteration:

Return to Step 2 and continue to complete the next loop until the iteration termination condition is met. The optimized parameters K are obtained, and the trajectory planning of the redundant manipulator is carried out by the PI control method. 

The Algorithm 1 implementation pseudocode of the PI control method with HGSO parameter regulator for trajectory planning is as follows:
**Algorithm 1** PI control method with HGSO parameter regulatorImport: trajectory points Output: PI parameters and tracking trajectory **Initialization:** Initialize HGSO parameters, N, M, $X_{i}$ (about PI controller parameters 4K), $H_{j}$, $P_{i,j}$, $C_{j}$, etc. using Equation (17) and Equation (18) (i=1,⋯,N; j=1,⋯M) **while**
*t* < the maximum number of iterations **do** Set the $X_{i}$ obtained by HGSO to PI parameters K for solving joint angles according to the part of PI controller. Complete the evaluation of the current $X_{i}$ by Equation (24). **for** each search gas particles **do** Update the position information $X_{i}$ using Equation (21). **for end** Update Henry’s coefficient of each gas type clusters using Equation (19). Update solubility of each gas using Equation (20) Select the poor particles according to Equation (22) by $F_{fitness}$. Reset the position of these worst particles by Equation (19) to Equation (21). *t* = *t* + 1 **while end** **return**
$X_{best}$ (The final optimized parameters K)

## 4. Simulation and Results

### 4.1. The Introduction of 9-DOF Hyper-Redundant Manipulator

**Figure 6:**
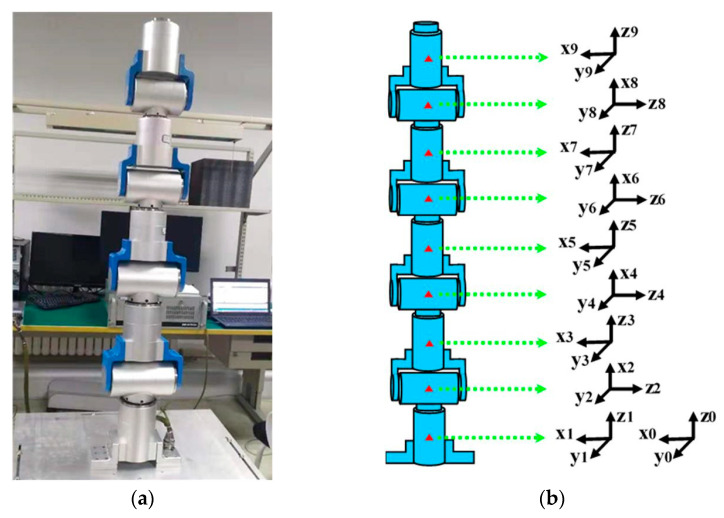
The correlogram of 9-DOF hyper-redundant manipulator. (**a**) Structure of the manipulator; (**b**) Establishment of coordinate system.

In this paper, based on the model of the 9-DOF hyper-redundant manipulator, the feasibility and advantages of the proposed PI-HGSO are verified. The structure of the 9-DOF hyper-redundant manipulator is shown in Figure 6a, and its rotation axis is assembled alternately at 90 degrees. At the same time, the screw method is adopted for kinematic modeling, and the centroid of each joint is established as the origin of the coordinates. The establishment of a coordinate system is shown in Figure 6b, and the related parameters are shown in Table 1.

### 4.2. The Introduction of 9-DOF Hyper-Redundant Manipulator

In the experimental part, the linear trajectory and the curve trajectory are taken as examples to prove the effectiveness of PI-HGSO by simulation. The related parameters of algorithms are listed in Table 2.

**Figure 7:**
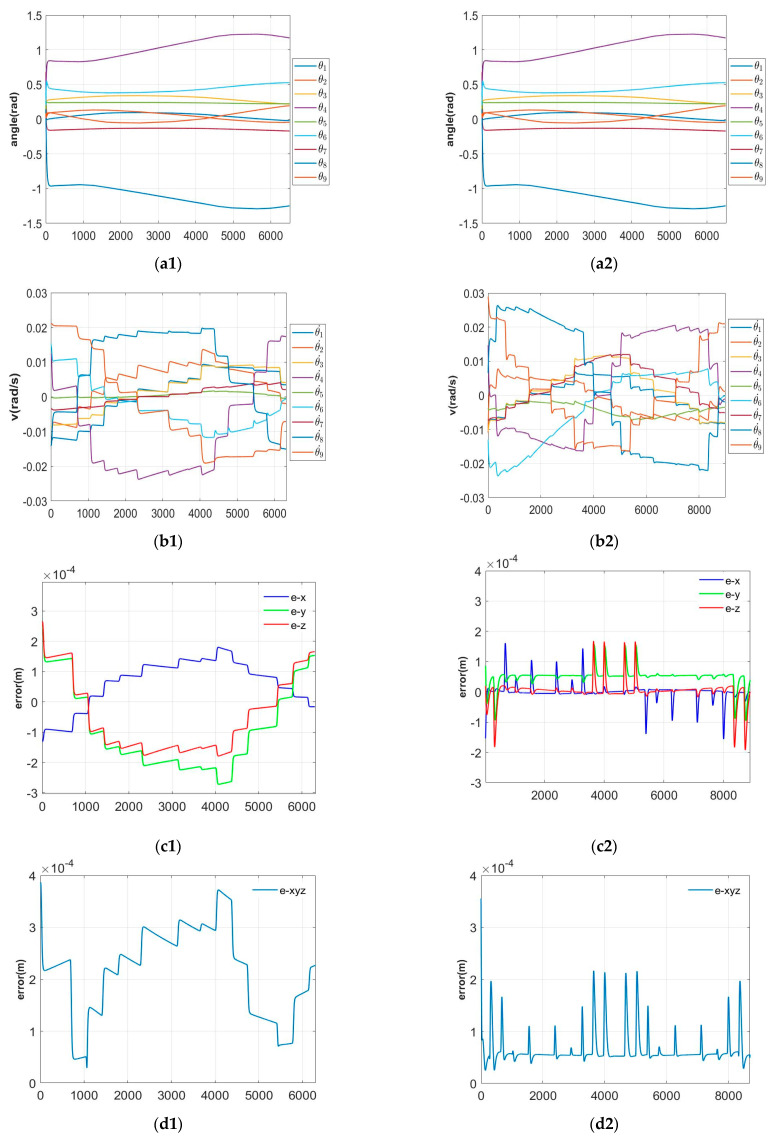

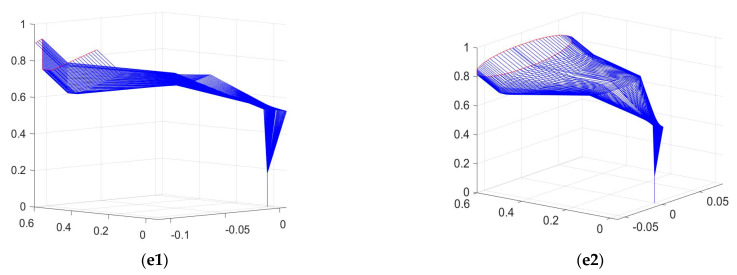
Simulations of the trajectory planning by PI-HGSO under lines and curves. (**a1**) Joint angles under line; (**a2**) Joint angles under curve; (**b1**) Joint velocities under line; (**b2**) Joint velocities under curve; (**c1**) Error in x, y, and z under line; (**c2**) Error in x, y, and z under curve; (**d1**) Error of end-effector under line; (**d2**) Error of end-effector under curve; (**e1**) Motion trajectories under line; (**e2**) Motion trajectories under curve.

The gain results of parameter optimization obtained by PI-HGSO are as follows: linear trajectory gain is $k_{p}=\left[\begin{array}{cc} 0.2784\times 10^{-4} & 30.6587 \end{array}\right]$, $k_{i}=\left[0.3399\right]$; curve trajectory gain is $k_{p}=\left[\begin{array}{cc} 174.7392 & 143.3465 \end{array}\right]$, $k_{i}=\left[2.9170\times 10^{3}\right]$. Figure 7 shows the trajectory planning results of the 9-DOF hyper-redundant manipulator through the linear trajectory and curve trajectory planning under PI-HGSO. At the same time, in order to further prove the advantageous performance superiorities of the designed PI-HGSO than others, the PI algorithm without parameter regulator is compared with the PI-HGSO. Furthermore, the PI-PSO, PI-GA, PI-ACO, and PI-GWO use particle swarm optimization algorithm, genetic algorithm, ant colony algorithm, and grey wolf optimizer to adjust the PI controller, are separately compared with the PI-HGSO. The simulation results are shown in Table 3. The effectiveness and superiority of PI-HGSO can be illustrated in Figure 7 and Table 3.

The trajectory planning results of the 9-DOF manipulator through the linear trajectory and curve trajectory planning are presented by PI-HGSO in Figure 7. What is more, Table 3 shows the comparison of PI-HGSO and other algorithms. It can be seen from Figure 7(a1,a2) that joint angles are all within joint limits, regardless of lines or curves. Meanwhile, the joint velocities are less than $2.5\times 10^{-2}$ rad/s and $3\times 10^{-2}$ rad/s, respectively, in Figure 7(b1,b2), it fully meets the joint velocity constraint of 0.1 rad/s. Figure 7(c1,c2) show the errors of the end effector on the x, y, and z axes, which are within $3\times 10^{-4}$ m and $2.5\times 10^{-4}$ m, respectively. At the same time, the vector two norm of the whole end effector errors serve as the overall end effector errors. It can be known that the error values are all under $4\times 10^{-4}$ m from Figure 7(d1,d2), and the errors are much smaller than the PI method, which adjusts parameters by experience in Table 3. Figure 7(e1,e2) are the motion trajectory simulation diagrams of the 9-DOF manipulator. In summary, it can be concluded that the trajectory planning errors are small, and the joint angles and joint velocities are within the limits by using PI-HGSO.

**Figure 8:**
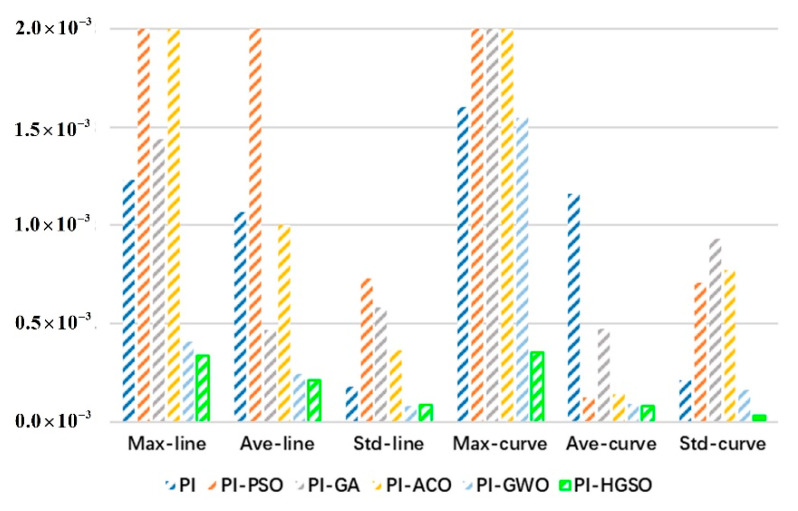
The comparative results of PI-HGSO and other algorithms about errors.

Furthermore, for each algorithm, 30 iterations of experiments were carried out, and corresponding data analysis values were obtained. As shown in Table 3 and Figure 8, the PI controller parameters optimized by the algorithms will be better than the result of manually adjusting the PI controller in average trajectory errors. The results of Wilson’s rank-sum test also show that the designed PI-HGSO has different average error values and is much better than PI. The experimental results are depicted in Table 3, the average errors of PI-HGSO are improved by 79.91% and 92.83%, respectively, under the test of line and curve trajectory than PI. This suggests that the errors are far less than that of manually adjusting PI and the trajectory planning effect is very good.

Meanwhile, it is obtained that the average errors of PI-HGSO are $2.15\times 10^{-4}$ m and $8.32\times 10^{-5}$ m, which are better than other comparison algorithms in Table 3 and Figure 8. The maximum errors of all trajectory points are $3.38\times 10^{-4}$ m and $3.55\times 10^{-4}$ m, which are the minimum in the comparison algorithms. In addition, the standard deviations are $8.47\times 10^{-5}$ m and $3.35\times 10^{-5}$ m, and the error changes of tracking points are small and stable in the whole trajectory tracking process. In the case of Table 3 and Figure 8, to sum up, PI-HGSO has a smaller average trajectory error, maximum error, and standard deviation of tracking points than PI, PI-PSO, PI-GA, PI-ACO, and PI-GWO. That is, the trajectory planning error and error fluctuation, which are obtained by PI-HGSO, are small, and the best trajectory planning of redundant manipulator can be achieved by PI-HGSO than others.

## 5. Experiment

**Figure 9:**
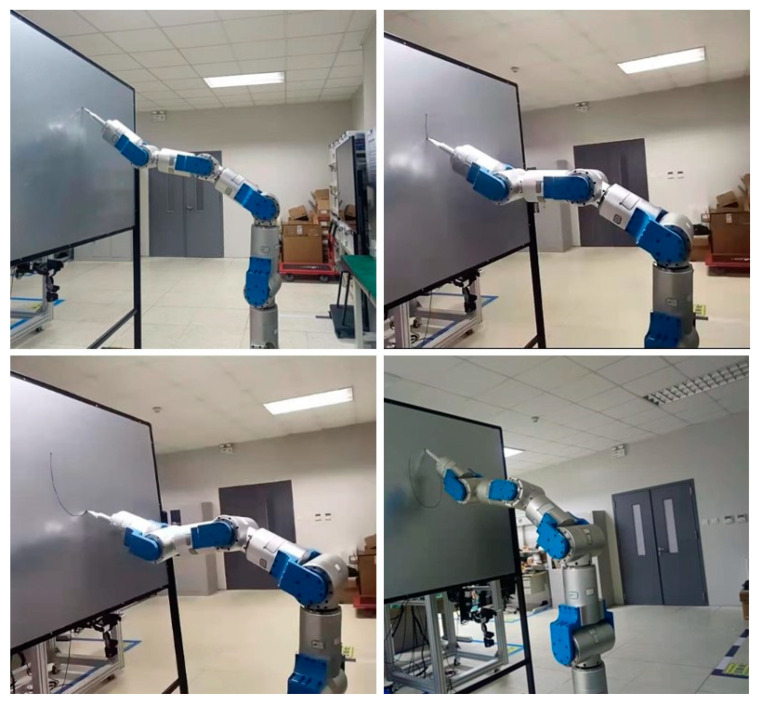
The experimental results of the PI-HGSO.

In order to complete the practical test of the proposed algorithm in this paper, the experimental verification of the PI-HGSO was carried out with the 9-DOF hyper-redundant manipulator entity of our laboratory. The above simulation data of the elliptic curve was used for the experiment, and the experimental trajectory result is shown in Figure 9. According to the experimental process and result, the joint angles are within the constraint range. Simultaneously, the joint velocities and the position tracking deviations are close to the simulation results. The results of each index meet the expected requirements, and the feasibility and superiority of the designed PI-HGSO are fully verified.

## 6. Conclusions

In this paper, a PI control method with HGSO parameter regulator for trajectory planning of 9-DOF redundant manipulator is proposed. In this method, the PI controller was selected to optimize the motion deviation of the manipulator end-effector. Then, the Jacobian matrix with weight coefficient is used to limit the joint angles. Meanwhile, HGSO was used to adjust PI controller parameters, and appropriate parameters were obtained to minimize the trajectory planning error. Experimental results demonstrate that the proposed method can not only obtain a small error value but also, the fluctuation of the error of each point is smaller than other algorithms. Besides, PI-HGSO can greatly improve the adjustment time and parameter accuracy compared with the original manual trial with experience.

However, there are some areas where this approach needs to be improved. For example, the iteration time of superior intelligent algorithms is limited, and the real-time performance after different trajectory changes cannot be realized. That could be the future research direction to realize the fast response of parameter adjustment.

## Figures and Tables

**Table 1:** The parameter table of screw modeling.

Joint i	Rotating Vector w	Translation Vector v (Unit: mm)
**1**	[0 0 1]	[0 0 0]
**2**	[1 0 0]	[−136.80 −0.10 5.33]
**3**	[0 0 1]	[13.66 −0.10 −286.11]
**4**	[1 0 0]	[−483.75 −0.10 4.85]
**5**	[0 0 1]	[16.26 −0.53 −621.91]
**6**	[1 0 0]	[−815.07 0.23 7.38]
**7**	[0 0 1]	[14.17 −0.50 −940.63]
**8**	[1 0 0]	[−1114.45 −0.31 6.24]
**9**	[0 0 1]	[10.92 −0.57 −1241.08]

**Table 2:** The Parameter settings of algorithms.

Algorithm	Parameters
**GA**	Population size N=50 Crossover probability α=0.8 Mutation probability β=0.5 Iterations Nc=50
**PSO**	Population size N=50 Inertia weight decreases linearly from 0.9 to 0.4 Individual-best acceleration factor C1=2 Global-best acceleration factor C2=2 Iterations Nc=50
**ACO**	Population size N=50 Pheromone trail factor α=1 Heuristic information factor β=5 Evaporation rate ρ=0.5 Iterations Nc=50
**GWO**	Population size N=50 Variable a decreases linearly from 2 to 0 Iterations Nc=50
**HGSO**	Population size N=50 Cluster number M=5 Influencing factor α = 1 Component parameter of interaction ability β=1 Iterations Nc=50

**Table 3:** The comparative results of PI-HGSO and other algorithms.

Type	Algorithm	The Value and Percent of Deviation					Wilcoxon Rank Sum Test
		Max		Ave		Std	
		Value	Variation	Value	Percent	Value	Value
**Line**	No PI	1.42 × 10^−3^	/	1.11 × 10^−3^	/	1.56 × 10^−4^	/
	PI	1.23 × 10^−3^	/	1.07 × 10^−3^	/	1.80 × 10^−4^	/
	PI-PSO	8.42 × 10^−3^	−7.19 × 10^−3^	2.75 × 10^−3^	61.09% ↘	7.32 × 10^−4^	+
	PI-GA	1.44 × 10^−3^	−0.21 × 10^−3^	4.67 × 10^−4^	56.35% ↗	5.83 × 10^−4^	+
	PI-ACO	5.60 × 10^−3^	−4.37 × 10^−3^	1.00 × 10^−3^	6.54% ↗	3.67 × 10^−4^	-
	PI-GWO	4.06 × 10^−4^	0.82 × 10^−3^	2.44 × 10^−4^	77.19% ↗	8.30 × 10^−5^	+
	**PI-HGSO**	**3.38 × 10^−4^**	**0.89 × 10^−3^**	**2.15 × 10^−4^**	**79.91% ↗**	**8.47 × 10^−5^**	**+**
**Curve**	No PI	1.61 × 10^−3^	/	1.12 × 10^−3^	/	1.87 × 10^−4^	/
	PI	1.60 × 10^−3^	/	1.16 × 10^−3^	/	2.11 × 10^−4^	/
	PI-PSO	4.86 × 10^−3^	−3.26 × 10^−3^	1.27 × 10^−4^	89.05% ↗	7.07 × 10^−4^	+
	PI-GA	5.53 × 10^−3^	−3.93 × 10^−3^	4.76 × 10^−4^	58.96% ↗	9.32 × 10^−4^	+
	PI-ACO	5.33 × 10^−3^	−3.73 × 10^−3^	1.40 × 10^−4^	87.93% ↗	7.72 × 10^−4^	+
	PI-GWO	1.55 × 10^−3^	0.05 × 10^−3^	8.99 × 10^−5^	92.22% ↗	1.61 × 10^−4^	+
	**PI-HGSO**	**3.55 × 10^−4^**	**1.25 × 10^−3^**	**8.32 × 10^−5^**	**92.83% ↗**	**3.35 × 10^−5^**	**+**
